# Association of lifesaving versus non-lifesaving extracranial surgery with long-term outcome after severe traumatic brain injury: a prospective CENTER-TBI cohort analysis

**DOI:** 10.1186/s13017-026-00713-y

**Published:** 2026-07-10

**Authors:** Angelo Guglielmi, Francesca Graziano, Anna Fornoni, Paolo Mangili, Edoardo Picetti, Marta Baggiani, Randall M. Chesnut, Gregory W. J. Hawryluk, Virginia F. J. Newcombe, Paola Rebora, Stefania Galimberti, Giuseppe Citerio, CENTER-TBI investigators and participants

**Affiliations:** 1 University of Pavia, PhD in Experimental Medicine, Pavia, Italy; 2 Anesthesia and Intensive Care Department 1, Fondazione IRCCS Policlinico San Matteo, Pavia, Italy; 3 Biostatistics and Clinical Epidemiology, Fondazione IRCCS San Gerardo dei Tintori, Monza, Italy; 4 NeuroIntensive Care Unit, Department Neuroscience, IRCCS Fondazione San Gerardo dei Tintori, Monza, Italy; 5 Department of Anesthesia and Intensive Care, Parma University Hospital, Parma, Italy; 6 Department of Neurological Surgery, University of Washington, Seattle, USA; 7 Department of Global Health, University of Washington, Seattle, USA; 8 Cleveland Clinic Lerner College of Medicine of Case Western Reserve University College of Medicine, Cleveland, OH USA; 9 Uniformed Services University, Bethesda, MD USA; 10 Brain Trauma Foundation, Palo Alto, CA USA; 11 Department of Medicine, Addenbrooke’s Hospital, University of Cambridge, Cambridge, UK; 12 Bicocca Bioinformatics Biostatistics and Bioimaging Center B4, School of Medicine and Surgery, University of Milano-Bicocca, Milan, Italy; 13 School of Medicine and Surgery, University of Milano-Bicocca, Milan, Italy

## Abstract

**Background:**

Patients with traumatic brain injury (TBI) frequently undergo extracranial surgery (ES). These procedures may be lifesaving (LS), such as haemorrhage or damage-control interventions, or non-lifesaving (NLS), including definitive fracture fixation or wound closure. Whether ES independently affects neurological recovery remains unclear.

**Methods:**

We analysed adult ICU patients with polytrauma and structural TBI (ISS ≥ 16, AIS Brain ≥ 3, and at least one extracranial injury as AIS ≥ 3) enrolled in the prospective multicentre CENTER-TBI cohort across 65 European sites. Predictors of extracranial surgery (ES) were evaluated using a cause-specific Cox proportional hazards model with competing risks, considering death before surgery as a competing event. LS and NLS categories were assigned by local investigators based on urgency. Associations between ES category (LS, NLS, or no-ES) and six-month unfavourable neurological outcome (GOSE ≤ 4) were assessed using multivariable logistic regression adjusted for IMPACT extended prognostic variables. Sensitivity analyses excluding patients who died within 48 h and secondary analysis in ICU survivors evaluated six-month outcome while minimizing immortal time bias from later NLS procedures.

**Results:**

Of 870 patients, 369 (42.4%) underwent ES: 168 (19.3%) LS and 201 (23.1%) NLS. LS patients were younger, had higher ISS, greater haemodynamic instability, more severe shock, and higher transfusion requirements compared with NLS and no-ES groups. Unadjusted six-month unfavourable outcome rates did not differ significantly (48.9% no-ES, 51.8% LS, 42.3% NLS; *p* = 0.2). LS remained associated with worse neurological outcome after adjustment (OR 1.86, 95% CI 1.15–3.01; *p* = 0.011), whereas NLS was not. Results remained consistent across sensitivity analyses, the multiple imputation model, and the secondary analysis.

**Conclusions:**

Emergency/damage-control extracranial surgery operationally classified as LS identified a phenotype of patients with greater extracranial injury burden and physiological instability, associated with worse long-term neurological outcome. This association should be interpreted as reflecting injury severity and treatment indication rather than a direct detrimental effect of surgery itself. NLS procedures were not independently associated with unfavourable recovery.

**Trial registration:**

ClinicalTrials.gov Identifiers: NCT02210221.

**Supplementary Information:**

The online version contains supplementary material available at https://doi.org/10.1186/s13017-026-00713-y.

## Introduction

Traumatic brain injury (TBI) is one of the most common and severe forms of trauma, often accompanied by extracranial injuries that complicate acute management and influence outcome [1–3]. The impact is particularly evident in polytrauma patients, where systemic insults such as hypoxemia, hypotension, anaemia, and coagulopathy interact with intracranial pathology to influence both acute survival and long-term recovery [1, 2]. In the Collaborative European NeuroTrauma Effectiveness Research in TBI (CENTER-TBI) study, more than one-third of patients sustained severe extracranial injury and, in the intensive care unit (ICU) stratum, this proportion exceeded 50% [4]. These patients frequently require extracranial surgery (ES).

A key unresolved issue is whether extracranial surgical interventions contribute causally to secondary brain injury through physiological instability or simply act as markers of systemic injury severity.

For some, the indication is immediate and emergency/damage-control in intent, here operationally classified as lifesaving (LS), such as emergency laparotomy or thoracotomy for bleeding control, endovascular haemorrhage control, or selected external fixation procedures performed in an urgent setting. For others, the indication is less urgent: definitive fracture fixation, spinal stabilization, or wound closure. These non-lifesaving (NLS) procedures are often postponed due to concerns that ES could worsen brain injury, prevent the detection of an intracranial haematoma or delay recovery [5, 6]. However, evidence regarding the neurological safety of extracranial surgery in the acute phase of TBI remains inconsistent [7–11]. Historical series raised concern that early fracture fixation could exacerbate secondary brain injury by inducing intraoperative hypotension, hypoxemia, blood loss, or increased intracranial pressure (ICP) [8–10, 12]. However, subsequent trauma literature has tempered the view that early fixation is intrinsically harmful [13], suggesting instead that timing should be guided by systemic and intracranial physiology rather than by fixed delay alone [8–10, 12, 14].

Moreover, most prior analyses have treated extracranial surgery in traumatic brain injury (TBI) as a binary exposure, grouping heterogeneous procedures together and emphasizing timing rather than surgical urgency or physiological context [8, 12]. This binary conceptualization assumes a uniform biological effect and may obscure important heterogeneity related to surgical urgency and physiological context.

In particular it remains unclear whether the worse outcomes reported are attributable to extracranial surgery itself or to the greater systemic injury severity that necessitates surgical intervention. Several observational studies have reported associations between extracranial surgical procedures and poorer outcomes in patients with severe traumatic brain injury; however, these findings are difficult to interpret causally because patients requiring surgery often have more severe systemic trauma and physiological derangement at baseline. Operative exposure may therefore act as a surrogate marker of polytrauma and physiological instability rather than representing an independent causal factor [2, 8, 9, 11, 14].

Moreover, few studies have incorporated detailed systemic physiological data that would allow surgical exposure to be disentangled from underlying injury severity [1, 5, 6, 14]. Most prior analyses have treated extracranial surgery as a binary exposure, thereby conflating urgent LS performed under conditions of physiological instability with discretionary procedures undertaken after stabilization [6, 8, 10]. This simplification limits causal interpretation and may obscure clinically meaningful heterogeneity in the relationship between extracranial surgery and long-term neurological outcome.

In this study, we characterize ICU patients with polytrauma and severe TBI undergoing ES, explicitly distinguishing between LS procedures and NLS procedures. We identify factors associated with the probability of undergoing ES and evaluate the association between LS and NLS interventions and six-month neurological outcome. By stratifying extracranial surgery according to urgency, we aim to redefine the interpretation of operative exposure in TBI. We hypothesize that LS extracranial surgery would identify a subgroup characterized by profound systemic injury severity and be associated with worse neurological outcome, whereas NLS procedures performed in stabilized patients would not be independently associated with unfavourable long-term recovery.

## Methods

### Study design and inclusion criteria

The CENTER-TBI study (clinicaltrials.gov NCT02210221, www.center-tbi.edu) is an international prospective observational study including TBI patients admitted to 65 centres in Europe. Details regarding design, methodology, and screening/enrolment have been previously described [4, 15]. The study (EC grant 602150) was conducted in accordance with applicable European and national regulations. Informed consent by the patients and/or the legal representative/next of kin was obtained for all patients recruited (see https://www.center-tbi.eu/project/ethical-approval).

We included adults (≥ 18 years) admitted to intensive care unit (ICU) with polytrauma, defined as ISS ≥ 16, AIS Brain ≥ 3, and at least one extracranial injury as AIS ≥ 3, with 6-month GOSE available. No additional exclusion criteria were applied beyond those defined in the CENTER-TBI core dataset.

Patients were then stratified in subgroups according to type of extracranial surgery performed. ES was defined as the first non-neurosurgical operative procedure performed during the index hospitalization. ES procedures were classified based on the clinical urgency of the procedure according to predefined study criteria and recorded by local investigators at the time of data entry. To enhance reproducibility, LS procedures were operationally defined as interventions performed to control active haemorrhage or prevent imminent physiological collapse, whereas NLS procedures included delayed or definitive surgical management not immediately required for survival. Thus, LS was used as an operational urgency-based category reflecting emergency or damage-control intent as recorded by local investigators. It should not be interpreted as evidence that each individual procedure independently prevented death. This distinction is particularly relevant for external fixation, which may be performed for heterogeneous indications, including haemorrhage control, damage-control orthopaedics, or urgent stabilization. Tracheostomy performed within 24 h was classified as LS only when recorded as the first urgent non-neurosurgical operative procedure; isolated tracheostomy performed after 24 h and percutaneous endoscopic gastrostomy were classified as no-ES.

The study was reported according to the Strengthening the Reporting of Observational Studies in Epidemiology (STROBE) guidelines.

### Data collection

Details regarding data collection and management within the CENTER-TBI study have been previously described [15]. Prospectively collected variables included demographics, comorbidities, and injury characteristics, including TBI mechanism and injury severity scores. Admission variables comprised neuroradiological features (Marshall CT classification [16]), post-resuscitation Glasgow Coma Scale (GCS) score, pupillary reactivity, Injury Severity Score (ISS) [17], and Abbreviated Injury Scale (AIS) by body region.

Physiological parameters included arterial blood gas values, hypotension (systolic blood pressure < 90 mmHg), hypoxemia (SpO_2_ < 90%), and laboratory data (e.g., creatinine, glucose, haemoglobin). Early biomarkers of brain injury measured within 48 h from admission included neuron-specific enolase (NSE), neurofilament light chain (NfL), S100 calcium-binding protein B (S100B), glial fibrillary acidic protein (GFAP), ubiquitin carboxy-terminal hydrolase L1 (UCH-L1), and tau protein [18].

Blood samples were centrifuged within 60 min and serum stored at − 80°C. NSE and S100B were analysed using Elecsys S100 and NSE assays, run on the e602 module of the Cobas 8000 modular analyser, Roche Diagnostics. GFAP, NFL, t-tau and UCH-L1 were analysed using Single Molecule Arrays-based Human Neurology 4-Plex B assay, run on the SR-X benchtop assay platform (Quanterix Corp., Lexington, MA). Complete details of the biomarker sampling protocol can be found in Czeiter et al. [19].

Severe extracranial injury was defined as AIS ≥ 3. Injury mechanisms were categorized as low- or high-energy TBI. Low-energy mechanisms included: (1) ground-level fall; (2) ground-level fall with head impact against an object; (3) direct impact to the head; and (4) combinations of these mechanisms. All other injury mechanisms or combinations were classified as high-energy TBI [4]. Shock class was derived according to the latest American College of Surgeons ATLS guidelines [20], incorporating base excess at admission as a discriminative variable [20–24] (Supplementary Tables ESM5); in cases of overlapping criteria, the highest severity class was assigned. Additional ICU variables included type and duration of extracranial and intracranial surgery, type and number of blood transfusions, need of vasopressor therapy, fluid therapy (total fluid input and weekly balance), ICP monitoring, duration of invasive mechanical ventilation and complications (e.g. ventilator associated pneumonia, dialysis, need of intracranial surgery) during the first ICU week. Mortality and GCS were recorded at ICU discharge. Six-month unfavourable neurological outcome (GOSE ≤ 4) was assessed by trained personnel through structured follow-up (face-to-face, telephone, or postal assessment) [25].

### Statistical analysis

Continuous variables are reported as mean with standard deviation (SD) or median with interquartile range (IQR), as appropriate according to their distribution. Categorical variables are presented as counts and percentages. Between-group comparisons (no-ES, LS and NLS) were performed using χ^2^ or Fisher tests for categorical variables and Kruskal–Wallis test for continuous variables, as appropriate. P-values from multiple comparisons were adjusted using the Benjamini–Hochberg false discovery rate (FDR) method.

To identify factors associated with undergoing ES, LS and NLS procedures, cause-specific hazard models based on Cox proportional hazards regression with competing risks were used, considering death before surgery as a competing event. Candidate predictors included variables significant in univariable analyses and/or considered clinically relevant based on prior literature and biological plausibility.

For the primary outcome analysis, six-month unfavourable neurological outcome (GOSE ≤ 4) was modelled using multivariable logistic regression. Extracranial surgery exposure was coded as a three-level categorical variable (no-ES as reference; lifesaving; NLS). Models were adjusted for covariates included in the IMPACT extended prognostic model to account for established predictors of TBI outcome [26].

To address potential immortal time bias, sensitivity analyses were performed excluding patients who died within 48 h from injury. Additionally, a secondary analysis was performed in patients discharged alive from the ICU (*n* = 726) to assess six-month neurological outcome while reducing immortal time bias from later NLS procedures. All analyses were performed using the complete cases, missing data were handled using Multiple Imputation by Chained Equations (MICE), generating five imputed datasets. Estimates were pooled according to Rubin’s rules. Model results are presented as odds ratios (ORs) for logistic regression models and hazard ratios (HRs) for Cox proportional hazards models, with 95% confidence intervals (CIs).

All statistical tests were two-sided with a significance level of 0.05. Analyses were performed using R software (version 4.4.1; R Foundation for Statistical Computing, Vienna, Austria).

## Results

### Study population and baseline characteristics

Of 4,509 patients enrolled in CENTER-TBI, 870 adult ICU patients met inclusion criteria for polytrauma with TBI and available six-month outcome. Among them, 369 (42.4%) underwent extracranial surgery (ES): 168 (19.3%) lifesaving (LS) and 201 (23.1%) non-lifesaving (NLS). The remaining 501 patients (57.6%) did not undergo extracranial surgery (no-ES) (Figure ESM1).

No-ES patients were older than those undergoing LS or NLS procedures (median 53 vs. 42.5 and 44 years, respectively; *p* < 0.001). They had a higher prevalence of Marshall CT score > 2 (46.1% vs. 23.1% LS and 28.1% NLS; *p* < 0.001), but lower overall trauma severity, with lower ISS (38 vs. 46.5 and 43; *p* < 0.001) and fewer ATLS severe shock classes III–IV (13.3% vs. 30.0% LS and 20.6% NLS; *p* < 0.001).

LS patients exhibited the most pronounced systemic compromise at presentation. They had the highest ISS, the greatest proportion of severe shock (ATLS III–IV), and the highest rates of hypotension (36.7%), emergency transfusion (22%), and coagulopathy correction (48.2%). In contrast, NLS patients showed intermediate injury severity and physiological instability. Additional baseline markers supported this profile: LS patients had lower MAP at arrival, lower first haemoglobin and fibrinogen values, and more negative base excess than the other groups, although lactate was limited by substantial missingness and did not differ significantly across groups (Table 1 and ESM1).

Early brain injury biomarkers further differentiated the groups: S100B and NSE concentrations were highest in LS patients, lowest in no-ES patients, and intermediate in NLS patients. Full baseline physiological and laboratory differences are reported in Table 1 and Supplementary Tables ESM1 and ESM2.

### Trauma characteristics and peri-trauma complications

High-energy trauma was most frequent in LS patients (99.4%), followed by NLS (94.5%) and no-ES (88.2%) (*p* < 0.001). Road traffic accidents predominated in LS (75.0%) and NLS (61.3%) compared with no-ES (50.7%), whereas incidental falls were more common in no-ES (39.3%) (*p* < 0.001).

Severe extracranial injuries (AIS ≥ 3) were more frequent in LS and NLS groups, particularly for extremities (58.1% LS; 51.2% NLS; 15.2% no-ES), abdomen/pelvis (50.0% LS; 39.3% NLS; 22.0% no-ES), and spine (36.9% LS; 43.3% NLS; 27.3% no-ES) (all *p* < 0.001), Supplementary Figure ESM2.

Neurological worsening in the emergency department was more frequent in no-ES patients (23.7%) than LS (14.0%) and NLS (17.4%) (*p* = 0.019). Hypotension (SBP < 90 mmHg) was most frequent in LS (36.7%), followed by NLS (26.0%) and no-ES (14.2%) (*p* < 0.001), Supplementary Table ESM1.

## Extracranial surgery characteristics and predictors

**Fig. 1:**
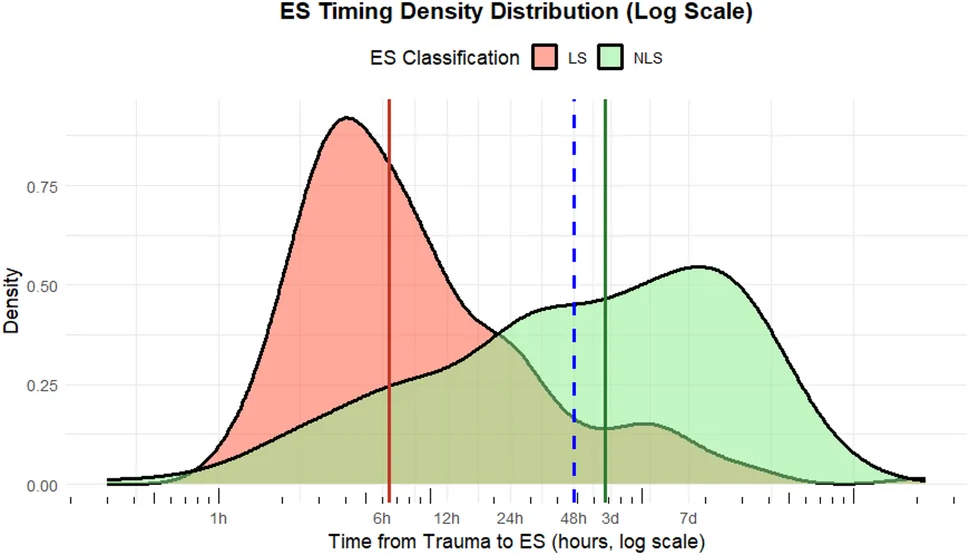
Timing of extracranial surgery according to urgency classification. Density distribution of the interval between traumatic brain injury and extracranial surgery (ES), stratified by urgency classification as life-saving ES (LS) or non-life-saving ES (NLS). Time from injury to surgery is displayed on a logarithmic scale. Solid vertical lines indicate the median time to surgery for each group. The dashed vertical line marks the 48-hour threshold after injury

Among the 369 ES procedures, the most frequent were internal fixation of extremity fractures (75/369, 20%), external fixation of extremity fractures (69/369, 19%), and spinal stabilization (46/369, 12%) (Supplementary Table ESM3). The LS category was clinically heterogeneous. Among 168 LS procedures, limb external fixation accounted for 37 cases (22%) and pelvic external fixation for 10 cases (6%), together representing 47/168 LS procedures (28%). Thus, external fixation was an important but not predominant component of the LS group. Other LS procedures included laparotomy in 21 patients (13%), thoracotomy in 3 (1.8%), endovascular treatment in 10 (6%), spinal stabilization in 15 (8.9%), limb internal fixation in 18 (11%), wound closure/graft in 10 (6%), tracheostomy within 24 h in 19 (11.3%) and other emergency procedures in 14 (8.3%). Overall, this distribution supports the interpretation of LS as an operational emergency/damage-control category rather than as a homogeneous surgical exposure. Median ES duration was 2.0 [1.3–3.2] hours. Timing differed markedly between groups: LS procedures were performed at 6.1 [3.3–15.3] hours from trauma, whereas NLS procedures were performed at 67.0 [18.8–205.8] hours (Fig. 1). Additional comparison of LS external fixation versus other LS procedures is reported in Supplementary Table ESM6.

**Fig. 2:**
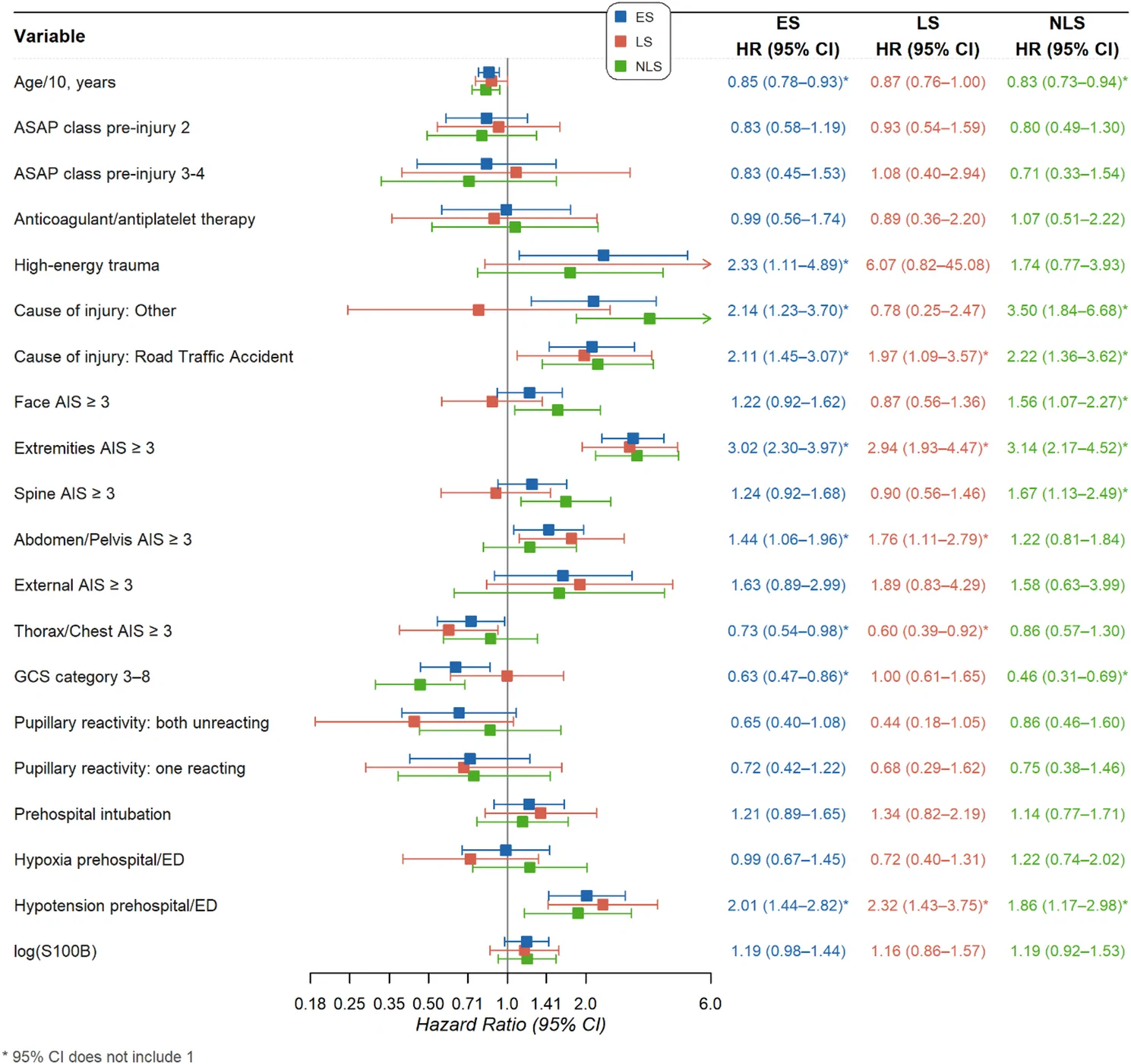
Predictors of extracranial surgery and urgency classification. Forest plot of cause-specific Cox regression models with death before surgery treated as a competing event. The models evaluate predictors of any extracranial surgery (ES), life-saving extracranial surgery (LS), and non-life-saving extracranial surgery (NLS). Cause-specific hazard ratios (HRs) are shown with 95% confidence intervals (CIs). Models were fitted in patients with complete covariate data (*n* = 512). The overall ES model included 222 events, while the LS and NLS models included 95 and 127 events, respectively. Error bars represent 95% CIs; statistical significance is indicated when the CI does not cross 1. ASAPS, American Society of Anesthesiologists Physical Status; AIS, Abbreviated Injury Scale; GCS, Glasgow Coma Scale; ED, Emergency Department

Predictors of ES included younger age, high-energy trauma mechanisms, road traffic accidents, multiple extracranial injuries involving the extremities, abdomen/pelvis, and thorax, severe neurological impairment (GCS 3–8), and prehospital/ED hypotension. However, when procedures were stratified by urgency, age and GCS category 3–8 were no longer predictors of LS surgery (Fig. 2), suggesting that LS classification was driven mainly by systemic injury burden and physiological instability rather than by neurological severity alone.

### Early ICU course

During the first ICU week, LS patients required the highest intensity of organ support, receiving greater total fluid volumes and markedly higher transfusion requirements—including red blood cells, plasma, and platelets—compared with both NLS and no-ES groups. Vasopressor use was also more frequent in LS patients (71.1%), while rates were similar in NLS (58.5%) and no-ES (58.3%) groups. RBC transfusion was required in 68.1% of LS patients, plasma transfusion in 43.4%, and platelet transfusion in 22.3%. Blood product exposure was also greatest in the LS group, including RBC transfusion, plasma transfusion, and platelet transfusion during the first ICU week (Table 2). In contrast, no-ES patients had the lowest transfusion requirements and overall support intensity. Rates of ICP monitoring and major neurological complications during the first week did not differ significantly across groups. Detailed ICU physiological and laboratory parameters are reported in Table 2.

**Table 1:** Baseline characteristics of ICU polytrauma patients stratified by extracranial surgery classification

		ES Status			
Variable	Overall *N* = 870^a^	No ES *N* = 501^a^	LS *N* = 168^a^	NLS *N* = 201^a^	q^c^
Sex female	232/870 (26.7%)	143/501 (28.5%)	41/168 (24.4%)	48/201 (23.9%)	0.4
Age, years	49.00 (29.00, 63.00)	53.00 (34.00, 67.00)	42.50 (27.00, 56.00)	44.00 (28.00, 58.00)	**< 0.001**
Frailty	0.07 (0.04, 0.14)	0.07 (0.03, 0.14)	0.07 (0.04, 0.11)	0.10 (0.04, 0.14)	0.2
Anticoagulants or Antiplatelets drugs	98/819 (12.0%)	71/468 (15.2%)	9/159 (5.7%)	18/192 (9.4%)	**0.007**
Pre-Injury ASAPS > 2	73/834 (8.8%)	47/476 (9.9%)	8/161 (5.0%)	18/197 (9.1%)	0.2
Missing	36	25	7	4	
At Arrival (Prehospital/ED)					
GCS					0.4
3–8	479/821 (58.3%)	290/475 (61.1%)	89/158 (56.3%)	100/188 (53.2%)	
9–12	110/821 (13.4%)	62/475 (13.1%)	20/158 (12.7%)	28/188 (14.9%)	
13–15	232/821 (28.3%)	123/475 (25.9%)	49/158 (31.0%)	60/188 (31.9%)	
Missing	49	26	10	13	
Pupillary reactivity					0.12
Both non reacting	118/831 (14.2%)	81/482 (16.8%)	15/158 (9.5%)	22/191 (11.5%)	
One reacting	72/831 (8.7%)	45/482 (9.3%)	11/158 (7.0%)	16/191 (8.4%)	
Missing	39	19	10	10	
CT: Marshall scale > 2	294/785 (37.5%)	208/451 (46.1%)	36/156 (23.1%)	50/178 (28.1%)	**< 0.001**
Missing	85	50	12	23	
Total ISS	41.00 (34.00, 50.00)	38.00 (32.00, 43.00)	46.50 (39.50, 57.00)	43.00 (34.00, 50.00)	**< 0.001**
ATLS shock class^b^					**< 0.001**
Class 1	570/823 (69.3%)	353/478 (73.8%)	97/160 (60.6%)	120/185 (64.9%)	
Class 2	103/823 (12.5%)	61/478 (12.8%)	15/160 (9.4%)	27/185 (14.6%)	
Class 3	14/823 (1.7%)	5/478 (1.0%)	7/160 (4.4%)	2/185 (1.1%)	
Class 4	136/823 (16.5%)	59/478 (12.3%)	41/160 (25.6%)	36/185 (19.5%)	
Missing	47	23	8	16	
MAP At Arrival, mmHg	95.00 (80.67, 108.67)	100.00 (86.67, 110.67)	86.67 (70.00, 97.50)	90.00 (77.67, 105.67)	**< 0.001**
Missing	49	23	8	18	
Heart rate at arrival, bpm	85.00 (73.00, 101.00)	83.00 (70.00, 99.00)	95.00 (80.00, 112.00)	88.00 (75.00, 107.50)	**< 0.001**
Missing	56	33	6	17	
pH	7.33 (7.27, 7.39)	7.35 (7.28, 7.39)	7.32 (7.23, 7.37)	7.32 (7.27, 7.38)	**0.008**
Missing	318	180	55	83	
Base excess, mmol/L	− 3.10 (− 6.00, − 1.00)	− 2.80 (− 5.70, − 0.80)	− 4.65 (− 7.25, − 1.45)	− 3.45 (− 5.40, − 1.10)	**0.026**
Missing	353	202	60	91	
First glucose, mg/dL	136.92 (117.10, 163.94)	134.04 (114.04, 162.14)	142.32 (119.98, 171.15)	134.04 (118.45, 156.74)	0.15
Missing	32	24	3	5	
First haemoglobin, g/dL	11.30 (9.60, 12.96)	11.52 (9.80, 13.28)	10.50 (9.20, 12.70)	10.80 (9.40, 12.55)	**0.002**
Missing	12	8	3	1	
First platelets count, U/mm^3^	178.00 (135.00, 221.00)	184.00 (145.00, 226.00)	175.00 (126.00, 211.00)	169.00 (131.00, 219.00)	**0.009**
Missing	20	15	3	2	
First fibrinogen, mg/dL	273.00 (200.00, 370.00)	280.00 (200.00, 363.00)	240.00 (174.00, 340.00)	281.50 (208.00, 400.00)	**0.011**
Missing	184	126	27	31	

Baseline demographic, clinical, radiological, and physiological characteristics stratified by extracranial surgery (ES) status: no ES, life-saving ES (LS), and non–life-saving ES (NLS)

^a^Continuous variables are presented as median (interquartile range [IQR]); categorical variables as n/N (%)

^b^Shock class was derived according to ATLS criteria; in cases of overlap, the highest severity class was assigned

^c^Group comparisons were performed using Kruskal–Wallis tests for continuous variables and χ^2^ tests for categorical variables, with Benjamini– Hochberg false discovery rate adjustment. P-values in bold indicate statistical significance (p < 0.05). Missing data proportions are reported. ED, emergency department; MAP, mean arterial pressure; CT, computed tomography; Pre-Injury ASAPS, pre-injury American Society of anesthesiologists physical status; GCS, glasgow coma scale; ISS, injury severity score; ICU, intensive care unit

### Outcomes

**Fig. 3:**
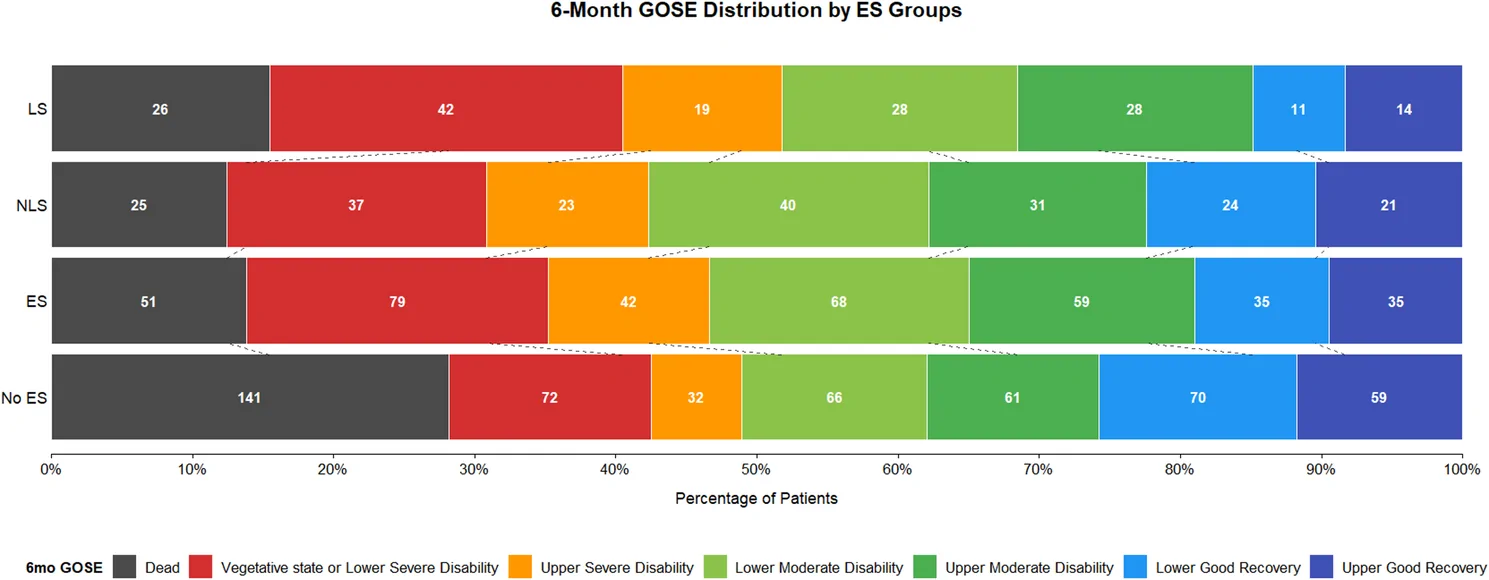
Six-month functional outcome according to extracranial surgery status. Distribution of Glasgow Outcome Scale–Extended (GOSE) categories at six months, stratified by extracranial surgery status: no ES, any ES, life-saving ES (LS), and non-life-saving ES (NLS). Bars show the proportion of patients in each GOSE category within each group. Unfavourable outcome was defined as GOSE ≤ 4. GOSE, Glasgow Outcome Scale–Extended; ES, extracranial surgery

ICU mortality was 21.8% in no-ES, 12.5% in LS, and 6.5% in NLS patients (*p* < 0.001) (Table 3; Fig. 3). Forty-eight–hour mortality was 7.6%, 4.2%, and 0.5%, respectively (*p* < 0.001). Withdrawal of treatment occurred in 15.8% of no-ES, 8.9% of LS, and 3.5% of NLS patients (*p* < 0.001). Six-month mortality was 28.1% in no-ES, 15.5% in LS, and 12.4% in NLS (*p* < 0.001).

**Table 2:** ICU resource utilization and physiological parameters during the first week

		ES status			
Variable	Overall *N* = 870^a^	No ES *N* = 501^a^	LS *N* = 168^a^	NLS *N* = 201^a^	q^b^
Total fluid input, litres	18.05 (8.06, 24.08)	16.16 (5.98, 22.35)	20.12 (11.94, 27.68)	19.98 (12.47, 25.85)	**< 0.001**
Missing	17	14	2	1	
Fluid balance, litres	0.45 (0.02, 0.94)	0.44 (0.01, 0.93)	0.58 (0.06, 1.19)	0.37 (-0.06, 0.80)	0.058
Missing	37	27	5	5	
Min haemoglobin, g/dL	9.00 (7.80, 10.50)	9.40 (8.10, 11.10)	8.40 (7.40, 9.40)	8.55 (7.51, 9.70)	**< 0.001**
Missing	10	6	3	1	
Min platelets, U/mm^b^	135.50 (101.00, 172.00)	149.00 (115.00, 181.00)	117.00 (90.00, 146.00)	124.00 (98.50, 157.50)	**< 0.001**
Missing	16	12	3	1	
Pneumonia	97/870 (11.1%)	50/501 (10.0%)	21/168 (12.5%)	26/201 (12.9%)	0.5
ICP Monitoring	452/865 (52.3%)	248/498 (49.8%)	97/167 (58.1%)	107/200 (53.5%)	0.2
ICP max value, first 48 h	19.00 (14.00, 25.00)	19.00 (15.00, 27.00)	19.00 (15.00, 24.00)	17.00 (13.00, 24.00)	
Missing	425	257	73	95	
ICP Mean Value, first 48 h	11.52 (8.35, 15.40)	11.95 (8.09, 16.31)	11.87 (9.09, 14.86)	10.08 (7.71, 14.50)	
Missing	425	257	73	95	
Emergency intracranial surgery					**< 0.001**
Craniotomy for haematoma/contusion	89/177 (50.3%)	73/132 (55.3%)	10/17 (58.8%)	6/28 (21.4%)	
Decompressive craniectomy	52/177 (29.4%)	40/132 (30.3%)	1/17 (5.9%)	11/28 (39.3%)	
Depressed skull fracture	14/177 (7.9%)	10/132 (7.6%)	3/17 (17.6%)	1/28 (3.6%)	
Other intracranial procedure	22/177 (12.4%)	9/132 (6.8%)	3/17 (17.6%)	10/28 (35.7%)	
Missing	1	1	0	0	
Vasopressors	519/853 (60.8%)	284/487 (58.3%)	118/166 (71.1%)	117/200 (58.5%)	**0.021**
Missing	17	14	2	1	
RBc transfusion	370/853 (43.4%)	154/487 (31.6%)	113/166 (68.1%)	103/200 (51.5%)	**< 0.001**
Missing	17	14	2	1	
Plasma transfusion	194/853 (22.7%)	66/487 (13.6%)	72/166 (43.4%)	56/200 (28.0%)	**< 0.001**
Missing	17	14	2	1	
Platelets transfusion	114/853 (13.4%)	42/487 (8.6%)	37/166 (22.3%)	35/200 (17.5%)	**< 0.001**
Missing	17	14	2	1	
Dialysis	21/853 (2.5%)	11/487 (2.3%)	4/166 (2.4%)	6/200 (3.0%)	0.8
Missing	17	14	2	1	

Organ support intensity, transfusion requirements, intracranial monitoring, and early ICU physiological variables during the first week of admission, stratified by extracranial surgery (ES) status: no ES, life-saving ES (LS), and non–life-saving ES (NLS). ^a^Continuous variables are expressed as median (IQR); categorical variables as n/N (%). Intracranial pressure (ICP) values refer to measurements within the first 48 h. Emergency intracranial procedures include craniotomy, decompressive craniectomy, depressed skull fracture repair, and other urgent neurosurgical interventions. ^b^Group comparisons were performed using Kruskal–Wallis tests for continuous variables and χ^2^ tests for categorical variables, with Benjamini– Hochberg false discovery rate adjustment. P-values in bold indicate statistical significance (*p* < 0.05). Missing data proportions are reported. RBC, red blood cells, ICU, Intensive Care Unit

Unfavourable neurological outcome at six months (GOSE ≤ 4) occurred in 48.9% of no-ES patients, 51.8% of LS patients, and 42.3% of NLS patients (*p* = 0.2). In multivariable analysis adjusted for IMPACT covariates (Table 4), LS surgery was independently associated with unfavourable six-month outcome (OR 1.86; 95% CI 1.15–3.01; *p* = 0.011), whereas NLS surgery was not (OR 0.87; 95% CI 0.55–1.36; *p* = 0.538). Results were consistent in the multiple imputation model (LS OR 1.66; *p* = 0.021), in sensitivity analysis excluding deaths within 48 h (LS OR 1.69; *p* = 0.018) and in the analysis restricted to patient discharged alive from the ICU (LS OR 2.42; *p* = 0.001).

**Table 3:** Short- and long-term clinical outcomes according to extracranial surgery classification

		ES Status			
Variable	Overall *N* = 870^a^	No ES *N* = 501^a^	LS *N* = 168^a^	NLS *N* = 201^a^	q^b^
Status at ICU discharge					**< 0.001**
Alive	726/870 (83.4%)	391/501 (78.0%)	147/168 (87.5%)	188/201 (93.5%)	
Dead	143/870 (16.4%)	109/501 (21.8%)	21/168 (12.5%)	13/201 (6.5%)	
Unknown	1/870 (0.1%)	1/501 (0.2%)	0/168 (0.0%)	0/201 (0.0%)	
48 h mortality	46/870 (5.3%)	38/501 (7.6%)	7/168 (4.2%)	1/201 (0.5%)	**< 0.001**
Withdrawal treatment in ICU	101/870 (11.6%)	79/501 (15.8%)	15/168 (8.9%)	7/201 (3.5%)	**< 0.001**
Timing withdrawal treatment in ICU, days	3.33 (1.03, 8.60)	3.11 (1.01, 7.64)	3.31 (1.18, 6.20)	12.65 (3.65, 19.75)	0.058
Missing	5	5	0	0	
Cause of death					0.058
Head injury/initial injury	79/121 (65.3%)	64/90 (71.1%)	6/15 (40.0%)	9/16 (56.3%)	
Head injury/secondary intracranial damage	21/121 (17.4%)	15/90 (16.7%)	4/15 (26.7%)	2/16 (12.5%)	
Systemic trauma	7/121 (5.8%)	3/90 (3.3%)	2/15 (13.3%)	2/16 (12.5%)	
Medical complications	7/121 (5.8%)	3/90 (3.3%)	2/15 (13.3%)	2/16 (12.5%)	
Other	7/121 (5.8%)	5/90 (5.6%)	1/15 (6.7%)	1/16 (6.3%)	
Missing	39	30	8	1	
6-mo mortality GOSE = 1	192/870 (22.1%)	141/501 (28.1%)	26/168 (15.5%)	25/201 (12.4%)	**< 0.001**
6-mo GOSE < = 4	417/870 (47.9%)	245/501 (48.9%)	87/168 (51.8%)	85/201 (42.3%)	0.2
In Hospital Length of stay (alive), days	21.39 (11.12, 37.24)	17.35 (8.00, 34.71)	27.57 (17.45, 47.31)	23.03 (13.92, 38.46)	**< 0.001**
Missing	22	13	6	3	
ICU Length of stay (alive), days	10.23 (3.18, 19.26)	7.92 (2.56, 17.17)	13.59 (5.79, 21.77)	11.79 (4.40, 20.57)	**< 0.001**
Missing	4	3	1	0	

ICU discharge status, early mortality, withdrawal of life-sustaining treatment, cause of death, length of stay, and six-month functional outcomes stratified by extracranial surgery (ES) status: no ES, life-saving ES (LS), and non–life-saving ES (NLS). ^a^Continuous variables are presented as median (IQR); categorical variables as n/N (%). Unfavourable outcome was defined as Glasgow Outcome Scale–Extended (GOSE) ≤ 4. ^b^Group comparisons were performed using Kruskal–Wallis tests for continuous variables and χ^2^ tests for categorical variables, with Benjamini– Hochberg false discovery rate adjustment. P-values in bold indicate statistical significance (*p* < 0.05). Missing data proportions are reported. ICU, intensive care unit, 6-mo, six months

**Table 4:** Multivariable logistic regression model for six-month functional outcome (GOSE)

Reference no ES		OR	Lower CI	Upper CI	*p*-value
MICE *N* = 870	NLS	1.101	0.740	1.639	0.632
	LS	1.658	1.079	2.547	**0.021**
Complete *N* = 696	NLS	0.868	0.553	1.362	0.538
	LS	1.860	1.150	3.009	**0.011**
Sensitivity (without 48 h dead) *N* = 824	NLS	1.189	0.801	1.766	0.390
	LS	1.685	1.094	2.594	**0.018**
Alive patients at ICU discharge (complete data) *N* = 587	NLS	1.313	0.821	2.097	0.255
	LS	2.423	1.449	4.052	**0.001**

Multivariable logistic regression assessing the association between extracranial surgery (ES) classification and six-month GOSE. The model was adjusted for IMPACT extended covariates (age, motor Glasgow Coma Scale, pupillary reactivity, hypoxia, hypotension, and CT characteristics). Extracranial surgery (ES) was categorized as lifesaving ES (LS) and non–life-saving ES (NLS) using no ES as the reference. Results are reported as odds ratios (OR) with 95% confidence intervals (CIs). P-values in bold indicate statistical significance (*p* < 0.05). Analyses included multiple imputation by chained equations (MICE), complete-case analysis, sensitivity analysis excluding patients who died within 48 hours and an analysis restricted to patients discharged alive from the ICU. ICU, intensive care unit

## Discussion

In this prospective multicentre analysis of ICU polytrauma patients with severe traumatic brain injury from the CENTER-TBI cohort, we evaluated the association between ES and six-month neurological outcome by explicitly distinguishing LS from NLS procedures.

The main finding of this study is that patients undergoing emergency/damage-control extracranial surgery operationally classified as LS had worse long-term neurological outcome, whereas patients undergoing NLS procedures did not. This should not be interpreted as evidence that LS surgery itself worsens neurological recovery. Rather, LS appears to identify a clinical phenotype characterized by greater extracranial injury burden, physiological instability, transfusion requirements, and urgent surgical indication. This association should be interpreted within the constraints of an observational design, where residual confounding by indication and time-related biases cannot be fully excluded.

Three findings are central. First, LS surgery identified a distinct physiological phenotype characterized by greater systemic derangement at presentation and higher subsequent resource requirements during the first ICU week. Second, after adjustment for established prognostic covariates included in the IMPACT extended model, LS classification remained statistically associated with unfavourable neurological outcome, whereas there was no evidence of an association between NLS surgery and six-month neurological outcome. Third, unadjusted rates of unfavourable outcome did not differ significantly across groups, underscoring the extent to which case-mix and physiological context shape crude comparisons. This reinforces the need to interpret crude outcome comparisons in light of case-mix, treatment indication, and competing risk. Importantly, these findings do not imply a detrimental effect of surgery itself but rather reflect the complex interplay between surgical indication and systemic injury severity.

The three study groups represent different clinical populations. These groups should not be viewed as interchangeable treatment arms. Patients without extracranial surgery were older, more frequently sustained low-energy mechanisms, had more severe intracranial imaging findings, and had higher rates of treatment limitation. Patients undergoing LS procedures were predominantly high-energy polytrauma patients with greater extracranial injury burden and more pronounced physiological derangement. Patients undergoing NLS procedures appeared to represent an intermediate phenotype in whom surgery was generally performed after initial stabilization.

### Phenotypic heterogeneity and interpretation of the adjusted association

In exploratory analyses, predictors of extracranial surgery included younger age, high-energy trauma mechanisms, road traffic accidents, the presence of multiple extracranial injuries (extremities, abdomen/pelvis, thorax), severe neurological impairment, and prehospital or emergency department hypotension. However, when extracranial procedures were stratified by urgency, age and GCS were no longer predictors of lifesaving surgery. This finding suggests that the need for LS procedures was driven primarily by the severity of systemic trauma and physiological instability rather than by neurological severity alone. Patients undergoing LS surgery exhibited the most pronounced markers of acute systemic compromise: higher injury severity scores, higher prevalence of ATLS shock classes III–IV, more frequent hypotension, worse metabolic acidosis, and higher early transfusion and coagulopathy-correction requirements. These differences persisted into early ICU care, with LS patients requiring greater fluid input, higher vasopressor use, and more frequent blood product transfusion. Early biomarker concentrations of neuronal injury (S100B and NSE) were highest in the LS group. In contrast, intracranial radiological severity, captured by Marshall CT > 2, was more prevalent in the no-ES group, indicating that LS classification primarily tracked systemic and extracranial burden rather than intracranial imaging severity alone.

Given this context, the independent association between LS surgery and unfavourable six-month outcome should be interpreted with caution. The analysis cannot establish causality, and residual confounding by indication is likely, as LS procedures are performed in patients with the most severe physiological instability and extracranial injury burden—factors not fully captured by conventional prognostic covariates. Thus, LS surgery primarily serves as a marker of extreme systemic threat and injury complexity rather than a direct cause of neurological deterioration.

An alternative interpretation of the differences observed between the no-ES and LS groups warrants consideration. Although patients undergoing LS procedures exhibited more pronounced early physiological derangement and higher levels of circulating biomarkers, this does not necessarily indicate a greater severity of primary brain injury at baseline. On the contrary, neuroimaging findings suggest that patients in the no-ES group may have had a higher burden of primary intracranial pathology.

A plausible explanation is that LS patients were exposed to a greater intensity of secondary systemic insults, such as hypotension, hypoxemia, and metabolic derangement, related to extracranial trauma, which may have influenced early neurological assessments and biomarker profiles. In this context, early indicators of neurological severity may partially reflect systemic injury and secondary insults rather than primary brain injury alone.

From a clinical standpoint, this distinction is relevant. In severely injured polytrauma patients, early neurological impairment may be overestimated, or even misattributed to primary brain injury, potentially influencing the perceived urgency and prioritization of lifesaving extracranial interventions [27–31] .

Taken together, these findings strongly support the interpretation of LS surgery as a surrogate marker of extreme systemic injury burden rather than an independent exposure affecting neurological outcome.

### Timing and procedural context: LS versus NLS

The timing of procedures supports this interpretation: LS procedures occurred substantially earlier after trauma than NLS procedures, consistent with their urgent indication. Procedure duration was similar across groups, indicating that urgency and physiological context, rather than operative time, were the key differences. External fixation deserves specific consideration. It is not a homogeneous exposure: in some patients, particularly those with pelvic disruption, severe open extremity trauma, or major extremity injury, external fixation may represent damage-control orthopaedics or contribute to haemorrhage control. In others, it may reflect urgent stabilization rather than an immediately lifesaving intervention. In our cohort, limb and pelvic external fixation accounted for 47/168 LS procedures (28%), while the remaining LS procedures included abdominal, thoracic, endovascular, spinal, internal fixation, wound, and other emergency procedures. This supports interpreting LS as an operational emergency/damage-control category rather than as a homogeneous biological exposure.

The distribution of extracranial injuries (AIS ≥ 3) also differed, with extremity, abdomen/pelvis, and spine injuries being substantially more frequent in LS and NLS groups than in the no-ES group, supporting the concept that ES reflects a broader polytrauma phenotype. NLS patients exhibited intermediate systemic compromise, and their early haemodynamic and laboratory profiles were closer to those of no-ES patients than to LS patients. After adjustment for IMPACT covariates, no evidence of an association was observed between NLS surgery and six-month neurological outcome. This finding aligns with contemporary evidence [14] suggesting that, in appropriately selected and stabilized patients, definitive extracranial procedures (such as orthopaedic fixation or stabilization) need not translate into an independent neurological penalty. Because each surgical procedure may affect cerebral physiology differently, this finding should not be interpreted as evidence of benefit or universal safety. Rather, it indicates absence of an independent association within the limits of this observational cohort. These comparisons are inherently susceptible to time-dependent biases, including immortal time bias, given the delayed occurrence of NLS procedures. This interpretation is consistent with the evolution of the fracture-fixation literature [13]. Historical concerns that early fixation could aggravate secondary brain injury through hypotension, hypoxemia, blood loss, or increased intracranial pressure have been tempered by subsequent trauma literature. Contemporary practice is better framed as physiology-guided timing rather than systematic delay. In patients without an immediate need for haemorrhage-control laparotomy/thoracotomy or emergent neurosurgical intervention, a pragmatic strategy may include repeat head CT within 12–24 h, treatment of intracranial hypertension, haemodynamic and metabolic resuscitation, and subsequent urgent fixation once systemic and intracranial physiology are acceptable.

### Relationship to prior literature and novelty

Prior investigations have often treated extracranial surgery as a binary exposure [8, 9, 11, 12], implicitly assuming a homogeneous biological effect and emphasizing timing rather than urgency and physiological context. Large contemporary analyses have reported associations between extracranial surgery and adverse longer-term outcomes [8, 12], but without stratification by clinical urgency. By contrast, our results suggest that the adverse prognostic signal is concentrated within patients requiring emergency/damage-control procedures operationally classified as LS, while there is no evidence of association between NLS procedures and unfavourable recovery after robust adjustment.

The novelty of this study lies in two elements. First, stratifying ES into LS and NLS categories exposes clinically meaningful heterogeneity that is obscured by binary definitions. Second, integrating early circulating biomarkers provides biological context: Although S100B and NSE were higher in LS patients, TBI severity did not differ between groups and Marshall scores were even higher in the no-ES group. Because both S100B and NSE may increase in the presence of extracranial injury, these findings may reflect differences in systemic trauma burden rather than intracranial injury severity alone. The absence of differences in more brain-enriched biomarkers such as GFAP and UCH-L1 further supports this interpretation [27–31]. These data do not demonstrate a surgical mechanism, but they strengthen the interpretation that LS surgery marks a state of profound physiological stress relevant to neurological prognosis.

### Mortality, withdrawal of treatment, and case-mix considerations

Mortality patterns further highlight case-mix complexity. In this cohort, ICU and six-month mortality were highest in the no-ES group, which was older, more frequently exposed to anticoagulants/antiplatelets, and had more severe intracranial imaging. The higher mortality observed in the no-ES group underscores that absence of extracranial surgery does not define a lower-risk population, but rather a distinct phenotype characterized by older age and greater intracranial imaging severity. Withdrawal of treatment in the ICU was also most frequent in the no-ES group. The no-ES group may comprise at least two distinct phenotypes: older adults with frailty, comorbidities, or treatment limitations, and patients with predominant intracranial injury in whom extracranial surgery was not indicated. These findings emphasize that the absence of extracranial surgery does not define a “less severe” population; rather, it may reflect a distinct phenotype characterized by older age and intracranial injury severity, where limitations of therapy are more frequent. Such patterns underscore why crude comparisons can be misleading and why interpretation should focus on adjusted estimates within clearly defined phenotypes. This apparent paradox reflects competing risk structures and differences in treatment limitation strategies.

### Strengths and limitations

This analysis leverages a large, prospectively collected multicentre cohort with standardized six-month outcome assessment, detailed systemic characterization, and early biomarker profiling. The use of IMPACT covariate adjustment, multiple imputation for missing data, sensitivity analyses excluding early deaths and analysis on ICU discharged alive patients only provides consistency across analytic approaches.

Several limitations merit consideration. First, the observational design precludes causal inference, and residual confounding by indication is likely, particularly for LS procedures, as the clinical decision to perform LS surgery is intrinsically driven by factors linked to outcome. From a causal perspective, extracranial surgery occurs downstream of injury severity and physiological instability, making it inherently vulnerable to both confounding by indication and potential collider bias.

Second, the classification of LS and NLS procedures was investigator-defined and may be subject to misclassification and inter-centre variability, potentially attenuating or distorting observed associations. This limitation is particularly relevant to external fixation. Detailed operative indications, fracture morphology, open versus closed fracture status, vascular injury, active bleeding, and surgeon-recorded rationale were not consistently available. Therefore, we could not distinguish external fixation performed primarily for haemorrhage control or damage-control orthopaedics from urgent stabilization in patients without overt haemodynamic instability. The term LS was retained to remain consistent with the clinician-assigned urgency variable available in the CENTER-TBI dataset. Third, NLS procedures occurred later and at variable time points during hospitalization; although sensitivity analyses excluding early deaths and analyses restricted to ICU survivors were performed to mitigate immortal time bias, modelling extracranial surgery as a time-dependent exposure would further strengthen causal interpretation.

Fourth, intraoperative physiological data were not available, precluding direct assessment of perioperative secondary insults, including intraoperative hypotension, hypoxemia, blood loss, transfusion requirements, ICP changes, and anesthetic management.

### Clinical implications

These findings refine the interpretation of extracranial operative exposure in severe TBI with polytrauma. Emergency/damage-control extracranial surgery operationally classified as LS identifies a phenotype of profound systemic compromise and worse long-term neurological recovery. NLS procedures, as performed in this cohort, showed no evidence of association with unfavourable outcome after adjustment. These data do not support delaying urgent damage-control procedures solely on the assumption that extracranial surgery is intrinsically harmful to neurological recovery, while decisions regarding NLS interventions should remain individualized and anchored to systemic stabilization and intracranial control rather than a uniform postponement paradigm.

## Conclusion

In polytrauma patients with severe traumatic brain injury, extracranial surgery identifies clinically distinct phenotypes rather than a uniform exposure. Emergency/damage-control procedures operationally classified as LS were associated with worse long-term neurological outcome, most likely reflecting systemic injury severity, physiological instability, and treatment indication rather than a direct deleterious surgical effect. In contrast, NLS procedures performed after initial stabilization showed no evidence of independent association with unfavourable recovery.

These findings support an individualized, physiology-guided interpretation of extracranial surgery in severe TBI. Future studies should adopt time-dependent and causal inference approaches, with detailed operative indications and intraoperative physiological data, to disentangle the independent effects of surgical interventions from underlying injury burden.

## Supplementary Information

Below is the link to the electronic supplementary material.

Supplementary Material 1.

Supplementary Material 2.

Supplementary Material 3.

## Data Availability

The data supporting the study findings are available upon reasonable request after approval of a proposal from the corresponding author (GC). Data collected for the analysis will be made available to others, including deidentified individual participant data and a data dictionary defining each field in the set. Related documents, such as the study protocol, statistical analysis plan, and informed consent form, will also be available.

## Abbreviations

ABG: Arterial blood gas
AIS: Abbreviated injury scale
aPTT: Activated partial thromboplastin time
ATLS: Advanced trauma life support
BE: Base excess
CENTER-TBI: Collaborative European NeuroTrauma Effectiveness Research in TBI
CI: Confidence interval
CT: Computed tomography
ED: Emergency department
ES: Extracranial surgery
FP7: Seventh framework programme
GCS: Glasgow coma scale
GFAP: Glial fibrillary acidic protein
GOSE: Glasgow outcome scale extended
Hb: Haemoglobin
HR: Hazard ratio
ICP: Intracranial pressure
ICU: Intensive care unit
INR: International normalized ratio
IQR: Interquartile range
ISS: Injury severity score
LS: Lifesaving extracranial surgery
MAP: Mean arterial pressure
MICE: Multiple imputation by chained equations
NfL: Neurofilament light chain
NLS: Non–lifesaving extracranial surgery
NSE: Neuron-specific enolase
OR: Odds ratio
PT: Prothrombin time
RBC: Red blood cells
S100B: S100 calcium-binding protein B
SD: Standard deviation
SpO_2_: Peripheral oxygen saturation
TBI: Traumatic brain injury
Tau: Tau protein
UCH-L1: Ubiquitin carboxy-terminal hydrolase L1

## References

[1] Watanabe T, Kawai Y, Iwamura A, Maegawa N, Fukushima H, Okuchi K. Outcomes after traumatic brain injury with concomitant severe extracranial injuries. Neurologia medico-chirurgica. 2018;58(9):393–9. 10.2176/nmc.oa.2018-0116. PMC6156128 30101808

[2] Carroll EL, Manktelow AE, Outtrim JG, et al. Influence of concomitant extracranial injury on functional and cognitive recovery from mild versus moderate to severe traumatic brain injury. J Head Trauma Rehab. 2020;35(6):E513–23. 10.1097/htr.0000000000000575. 32472833

[3] Maas AIR, Menon DK, Manley GT et al. Traumatic brain injury: progress and challenges in prevention, clinical care, and research. *Lancet Neurol*. 2022;21(Neurosurg Rev 43 2020):1004–1060. 10.1016/s1474-4422(22)00309-x PMC10427240 36183712

[4] Steyerberg EW, Wiegers E, Sewalt C, et al. Case-mix, care pathways, and outcomes in patients with traumatic brain injury in CENTER-TBI: a European prospective, multicentre, longitudinal, cohort study. Lancet Neurol. 2019;18(10):923–34. 10.1016/s1474-4422(19)30232-7. 31526754

[5] Moore JM, Thomas PAW, Gruen RL, Chan P, Rosenfled JV. Simultaneous multisystem surgery: an important capability for the civilian trauma hospital. Clin Neurol Neurosurg. 2016;148:13–6. 10.1016/j.clineuro.2016.06.012. 27359088

[6] Wang MC, Temkin NR, Deyo RA, Jurkovich GJ, Barber J, Dikmen S. Timing of surgery after multisystem injury with traumatic brain injury: effect on neuropsychological and functional outcome. J Trauma: Inj Infect Crit Care. 2007;62(5):1250–8. 10.1097/01.ta.0000215581.50234.56. 17495732

[7] Picetti E, Rossi S, Abu-Zidan FM et al. WSES consensus conference guidelines: monitoring and management of severe adult traumatic brain injury patients with polytrauma in the first 24 hours. World J Emerg Surg: WJES. 2019;14(1):53. 10.1186/s13017-019-0270-1 PMC6884766 31798673

[8] Roberts CJ, Barber J, Temkin NR, et al. Clinical outcomes after traumatic brain injury and exposure to extracranial surgery. JAMA Surg. 2024;159(3):248–59. 10.1001/jamasurg.2023.6374. PMC10719833 38091011

[9] Algarra NN, Lele AV, Prathep S, et al. Intraoperative secondary insults during orthopedic surgery in traumatic brain injury. J Neurosurg Anesthesiol. 2017;29(3):228–35. 10.1097/ana.0000000000000292. PMC5011033 26954768

[10] Jaicks RR, Cohn SM, Moller BA. Early fracture fixation may be deleterious after head injury. J Trauma: Inj Infect Crit Care. 1997;42(1):1–5. 10.1097/00005373-199701000-00001. 9003250

[11] Fujita Y, Algarra NN, Vavilala MS, Prathep S, Prapruettham S, Sharma D. Intraoperative secondary insults during extracranial surgery in children with traumatic brain injury. Child’s Nerv Syst. 2014;30(7):1201–8. 10.1007/s00381-014-2353-3. 24429505

[12] Roberts CJ, Maiga AW, Barber J, et al. One-year outcomes after traumatic brain injury and early extracranial surgery in the TRACK-TBI study. JAMA Netw Open. 2025;8(10):e2537271. 10.1001/jamanetworkopen.2025.37271. PMC12514633 41071547

[13] Scalea TM. Optimal timing of fracture fixation: Have we learned anything in the past 20 years? J Trauma: Inj Infect Crit Care. 2008;65(2):253–60. 10.1097/ta.0b013e31817fa475. 18695459

[14] Zheng J, Ouyang Y, Zhang K, et al. Early vs late fixation of extremity fractures among adults with traumatic brain injury. JAMA Netw Open. 2024;7(3):e241556. 10.1001/jamanetworkopen.2024.1556. PMC10924246 38457181

[15] Maas AIR, Menon DK, Steyerberg EW, et al. Collaborative European NeuroTrauma effectiveness research in traumatic brain injury (CENTER-TBI): a prospective longitudinal observational study. Neurosurgery. 2015;76(1):67–80. 10.1227/neu.0000000000000575. 25525693

[16] Marshall LF, Marshall SB, Klauber MR, et al. The diagnosis of head injury requires a classification based on computed axial tomography. J neurotrauma. 1992;9(Suppl 1):S287–92. 1588618

[17] Baker SP, O’neill B, Haddon W, Long WB. The injury severity score. J Trauma: Inj Infect Crit Care. 1974;14(3):187–96. 10.1097/00005373-197403000-00001. 4814394

[18] Hossain I, Marklund N, Czeiter E, Hutchinson P, Buki A. Blood biomarkers for traumatic brain injury: a narrative review of current evidence. Brain Spine. 2024;4:102735. 10.1016/j.bas.2023.102735. PMC10951700 38510630

[19] Czeiter E, Amrein K, Gravesteijn BY, et al. Blood biomarkers on admission in acute traumatic brain injury: relations to severity, CT findings and care path in the CENTER-TBI study. EBioMedicine. 2020;56:102785. 10.1016/j.ebiom.2020.102785. PMC7251365 32464528

[20] American College of Surgeons. ATLS: advanced trauma life support student course manual. 11– ed. ed. Chicago: American College of Surgeons; 2024.

[21] Mutschler M, Nienaber U, Brockamp T, et al. A critical reappraisal of the ATLS classification of hypovolaemic shock: Does it really reflect clinical reality? Resuscitation. 2013;84(3):309–13. 10.1016/j.resuscitation.2012.07.012. 22835498

[22] Davis JW, Sue LP, Dirks RC, et al. Admission base deficit is superior to lactate in identifying shock and resuscitative needs in trauma patients. Am J Surg. 2020;220(6):1480–4. 10.1016/j.amjsurg.2020.10.005. 33046221

[23] Kortbeek JB, Turki SAA, Ali J, et al. Advanced trauma life support, 8th Edition, the evidence for change. J Trauma: Inj Infect Crit Care. 2008;64(6):1638–50. 10.1097/ta.0b013e3181744b03. 18545134

[24] Guly HR, Bouamra O, Little R, et al. Testing the validity of the ATLS classification of hypovolaemic shock. Resuscitation. 2010;81(9):1142–7. 10.1016/j.resuscitation.2010.04.007. 20619954

[25] Wilson JTL, Pettigrew LEL, Teasdale GM. Structured interviews for the glasgow outcome scale and the extended glasgow outcome scale: guidelines for their use. J Neurotraum. 1998;15(8):573–85. 10.1089/neu.1998.15.573. 9726257

[26] Steyerberg EW, Mushkudiani N, Perel P, et al. Predicting outcome after traumatic brain injury: development and international validation of prognostic scores based on admission characteristics. Plos Med. 2008;5(8):e165. 10.1371/journal.pmed.0050165. discussion e165. PMC2494563 18684008

[27] Posti JP, Hossain I, Takala RSK, et al. Glial fibrillary acidic protein and ubiquitin C-terminal hydrolase-L1 are not specific biomarkers for mild CT-negative traumatic brain injury. J Neurotrauma. 2017;34(7):1427–38. 10.1089/neu.2016.4442. 27841729

[28] Müller M, Münster JM, Hautz WE, et al. Increased S-100 B levels are associated with fractures and soft tissue injury in multiple trauma patients. Injury. 2020;51(4):812–8. 10.1016/j.injury.2020.03.012. 32192718

[29] Pelinka LE, Hertz H, Mauritz W, et al. Non specific increase of systemic neuron-specific enolase after trauma: clinical and experimental findings. Shock. 2005;24(2):119–23. 10.1097/01.shk.0000168876.68154.43. 16044081

[30] Thelin EP, Jeppsson E, Frostell A, et al. Utility of neuron-specific enolase in traumatic brain injury; relations to S100B levels, outcome, and extracranial injury severity. Crit Care. 2016;20(1):285. 10.1186/s13054-016-1450-y. PMC5015335 27604350

[31] Anderson RE, Hansson LO, Nilsson O, Dijlai-Merzoug R, Settergren G. High Serum S100B Levels for Trauma Patients without Head Injuries. Neurosurgery. 2001;48(6):1255. 10.1097/00006123-200106000-00012. 11383727

