# Supplementary material for: Association of lifesaving versus non-lifesaving extracranial surgery with long-term outcome after severe traumatic brain injury: a prospective CENTER-TBI cohort analysis
Source: World J Emerg Surg. 2026 Jul 10;21:53. doi: 10.1186/s13017-026-00713-y (PMC13637195; doi:10.1186/s13017-026-00713-y)
Supplement: Supplementary file 2 [file 13017_2026_713_MOESM1_ESM.docx]

**Electronic Supplementary Material - ESM 1**

**Association of lifesaving versus non-lifesaving extracranial surgery with long-term outcome after severe traumatic brain injury: a prospective CENTER-TBI cohort analysis**

Angelo Guglielmi^1-2^, Francesca Graziano^3^, Anna Fornoni^4^, Paolo Mangili^4^, Edoardo Picetti^5^, Marta Baggiani^4^, Randall M. Chesnut^6-7^, Gregory W.J. Hawryluk^8,9,10^, Virginia F.J. Newcombe^11^, Paola Rebora^3,12^, Stefania Galimberti^3,12^, Giuseppe Citerio^4,13^; CENTER-TBI investigators and participants.

^1^University of Pavia, PhD in Experimental Medicine, Pavia, Italy.

^2^Anesthesia and Intensive Care Department 1, Fondazione IRCCS Policlinico San Matteo, Pavia, Italy.

^3^Biostatistics and Clinical Epidemiology, Fondazione IRCCS San Gerardo dei Tintori, Monza, Italy.

^4^NeuroIntensive Care Unit, Department Neuroscience, IRCCS Fondazione San Gerardo dei Tintori, Monza, Italy.

^5^Department of Anesthesia and Intensive Care, Parma University Hospital, Parma, Italy.

^6^Department of Neurological Surgery, University of Washington, Seattle, USA.

^7^Department of Global Health, University of Washington, Seattle, USA.

^8^Cleveland Clinic Lerner College of Medicine of Case Western Reserve University College of Medicine, Cleveland, Ohio, USA.

^9^Uniformed Services University, Bethesda, Maryland, USA.

^10^Brain Trauma Foundation, Palo Alto, California, USA.

^11^Department of Medicine, University of Cambridge, Addenbrooke's Hospital, Cambridge, UK.

^12^Bicocca Bioinformatics Biostatistics and Bioimaging Center B4, School of Medicine and Surgery, University of Milano-Bicocca, Milan, Italy.

^13^School of Medicine and Surgery, University of Milano-Bicocca, Milan, Italy.

Corresponding Author:

Prof. Giuseppe Citerio,

School of Medicine and Surgery, University of Milano-Bicocca, Milan, Italy.

Email: giuseppe.citerio@unimib.it

|  |  | **ES Status** | | |  |
| --- | --- | --- | --- | --- | --- |
| **Variable** | **Overall** N = 870^1^ | **No ES** N = 501^1^ | **LS** N = 168^1^ | **NLS** N = 201^1^ | **q**^3^ |
| **GCS Motor** |  |  |  |  | **0.4** |
| None | 343/853 (40.2%) | 206/493 (41.8%) | 68/165 (41.2%) | 69/195 (35.4%) |  |
| Abnormal extension | 28/853 (3.3%) | 19/493 (3.9%) | 5/165 (3.0%) | 4/195 (2.1%) |  |
| Abnormal flexion | 39/853 (4.6%) | 24/493 (4.9%) | 9/165 (5.5%) | 6/195 (3.1%) |  |
| Normal flexion/withdrawal | 61/853 (7.2%) | 39/493 (7.9%) | 8/165 (4.8%) | 14/195 (7.2%) |  |
| Localizes/obeys | 382/853 (44.8%) | 205/493 (41.6%) | 75/165 (45.5%) | 102/195 (52.3%) |  |
| Missing | 17 | 8 | 3 | 6 |  |
| **Intubation** | 469/862 (54.4%) | 266/498 (53.4%) | 100/167 (59.9%) | 103/197 (52.3%) | **0.3** |
| Missing | 8 | 3 | 1 | 4 |  |
| **Simultaneous extracranial and intracranial surgery** | 26/868 (3.0%) | 0/501 (0.0%) | 14/167 (8.4%) | 12/200 (6.0%) |  |
| Missing | 2 | 0 | 1 | 1 |  |
| **TRAUMA CHARATERISTICS** | | | | | |
| **Trauma Energy** |  |  |  |  | **<0.001** |
| High | 744/811 (91.7%) | 418/474 (88.2%) | 153/154 (99.4%) | 173/183 (94.5%) |  |
| Low | 67/811 (8.3%) | 56/474 (11.8%) | 1/154 (0.6%) | 10/183 (5.5%) |  |
| Missing | 59 | 27 | 14 | 18 |  |
| **Cause of Injury** |  |  |  |  | **<0.001** |
| Incidental Fall | 262/852 (30.8%) | 192/489 (39.3%) | 25/164 (15.2%) | 45/199 (22.6%) |  |
| Road Traffic Accident | 493/852 (57.9%) | 248/489 (50.7%) | 123/164 (75.0%) | 122/199 (61.3%) |  |
| Other | 97/852 (11.4%) | 49/489 (10.0%) | 16/164 (9.8%) | 32/199 (16.1%) |  |
| Missing | 18 | 12 | 4 | 2 |  |
| **Face (AIS≥3)** | 352/870 (40.5%) | 191/501 (38.1%) | 68/168 (40.5%) | 93/201 (46.3%) | 0.2 |
| **Extremities (AIS≥3)** | 276/869 (31.8%) | 76/501 (15.2%) | 97/167 (58.1%) | 103/201 (51.2%) | **<0.001** |
| Missing | 1 | 0 | 1 | 0 |  |
| **Spine^2^ (AIS≥3)** | 286/870 (32.9%) | 137/501 (27.3%) | 62/168 (36.9%) | 87/201 (43.3%) | **<0.001** |
| **Abdomen/Pelvis (AIS≥3)** | 273/870 (31.4%) | 110/501 (22.0%) | 84/168 (50.0%) | 79/201 (39.3%) | **<0.001** |
| **External wounds (AIS≥3)** | 46/870 (5.3%) | 16/501 (3.2%) | 16/168 (9.5%) | 14/201 (7.0%) | **0.007** |
| **Thorax/Chest (AIS≥3)** | 575/870 (66.1%) | 324/501 (64.7%) | 112/168 (66.7%) | 139/201 (69.2%) | 0.6 |
| **PERI-TRAUMA COMPLICATIONS** | | | | | |
| **Neurological Worsening (ED)** | 169/836 (20.2%) | 108/455 (23.7%) | 22/157 (14.0%) | 39/224 (17.4%) | **0.019** |
| Missing | 34 | 23 | 7 | 4 |  |
| **Hypoxemia, (SpO2 < 90%)** | 153/815 (18.8%) | 76/469 (16.2%) | 40/157 (25.5%) | 37/189 (19.6%) | 0.053 |
| Missing | 55 | 32 | 11 | 12 |  |
| **Hypotension (SBP < 90 mmHg)** | 175/821 (21.3%) | 67/471 (14.2%) | 58/158 (36.7%) | 50/192 (26.0%) | **<0.001** |
| Missing | 49 | 30 | 10 | 9 |  |
| **Hypothermia (Prehospital/ED)** | 106/778 (13.6%) | 64/447 (14.3%) | 21/149 (14.1%) | 21/182 (11.5%) | 0.7 |
| Missing | 92 | 54 | 19 | 19 |  |
| **Cardiac Arrest (ED)** | 38/865 (4.4%) | 22/498 (4.4%) | 12/167 (7.2%) | 4/200 (2.0%) | 0.082 |
| Missing | 5 | 3 | 1 | 1 |  |
| **Seizures (ED), Yes** |  |  |  |  | 0.2 |
| No | 799/832 (96.0%) | 450/474 (94.9%) | 161/162 (99.4%) | 188/196 (95.9%) |  |
| Partial/Focal | 14/832 (1.7%) | 11/474 (2.3%) | 0/162 (0.0%) | 3/196 (1.5%) |  |
| Generalized | 19/832 (2.3%) | 13/474 (2.7%) | 1/162 (0.6%) | 5/196 (2.6%) |  |
| Missing | 38 | 27 | 6 | 5 |  |
| **HAEMODYNAMICS and RESPIRATORY** | | | | | |
| **SBP at Arrival (mmHg)** | 130.00 (113.00, 150.00) | 137.50 (120.00, 155.00) | 120.00 (98.00, 135.00) | 126.00 (110.00, 146.00) | **<0.001** |
| Missing | 45 | 23 | 7 | 15 |  |
| **Temperature (°C)** | 36.00 (35.30, 36.70) | 36.00 (35.10, 36.60) | 36.20 (35.40, 36.80) | 36.10 (35.40, 36.70) | 0.4 |
| Missing | 437 | 242 | 89 | 106 |  |
| **Lactate (mmol/L)** | 2.20 (1.40, 3.60) | 2.20 (1.40, 3.50) | 2.40 (1.60, 3.95) | 2.20 (1.48, 3.70) | 0.5 |
| Missing | 376 | 213 | 64 | 99 |  |
| **Respiratory Rate** | 16.00 (14.00, 20.00) | 16.00 (14.00, 19.00) | 16.00 (14.00, 20.00) | 16.00 (14.00, 20.00) | 0.7 |
| Missing | 330 | 187 | 63 | 80 |  |
| **SpO2 (%)** | 99.00 (96.00, 100.00) | 99.00 (96.00, 100.00) | 99.00 (96.00, 100.00) | 99.00 (96.00, 100.00) | 0.6 |
| Missing | 90 | 41 | 19 | 30 |  |
| **TREATMENTS** |  |  |  |  |  |
| **Vasopressors (ED), Yes** | 169/869 (19.4%) | 83/500 (16.6%) | 34/168 (20.2%) | 52/201 (25.9%) | **0.034** |
| Missing | 1 | 1 | 0 | 0 |  |
| **IV Fluids (ED)** | 668/869 (76.9%) | 379/500 (75.8%) | 140/168 (83.3%) | 149/201 (74.1%) | 0.12 |
| Missing | 1 | 1 | 0 | 0 |  |
| Crystalloids | 540/869 (62.1%) | 295/500 (59.0%) | 121/168 (72.0%) | 124/201 (61.7%) |  |
| Missing | 1 | 1 | 0 | 0 |  |
| Mannitol | 70/869 (8.1%) | 49/500 (9.8%) | 10/168 (6.0%) | 11/201 (5.5%) |  |
| Missing | 1 | 1 | 0 | 0 |  |
| Saline | 78/869 (9.0%) | 48/500 (9.6%) | 14/168 (8.3%) | 16/201 (8.0%) |  |
| Missing | 1 | 1 | 0 | 0 |  |
| Colloids | 37/869 (4.3%) | 18/500 (3.6%) | 15/168 (8.9%) | 4/201 (2.0%) |  |
| Missing | 1 | 1 | 0 | 0 |  |
| Albumin | 9/869 (1.0%) | 2/500 (0.4%) | 3/168 (1.8%) | 4/201 (2.0%) |  |
| Missing | 1 | 1 | 0 | 0 |  |
| **Blood Transfusion** | 81/869 (9.3%) | 24/500 (4.8%) | 37/168 (22.0%) | 20/201 (10.0%) | **<0.001** |
| Missing | 1 | 1 | 0 | 0 |  |
| **Coagulopathy Correction (ED)** | 220/849 (25.9%) | 86/488 (17.6%) | 80/166 (48.2%) | 54/195 (27.7%) | **<0.001** |
| Missing | 21 | 13 | 2 | 6 |  |

## Supplementary Table ESM1. Arrival Characteristics and Emergency Department Treatments.

Vital signs, laboratory parameters, and emergency department treatments at presentation stratified by extracranial surgery (ES) status: no ES, lifesaving ES (LS), and nonlife-saving ES (NLS). ^1^Continuous variables are presented as median (IQR); categorical variables as n/N (%). ^2^Includes cervical spine, thoracic spine, lumbar spine. ^3^Group comparisons were performed using Kruskal–Wallis tests for continuous variables and χ² tests for categorical variables, with Benjamini– Hochberg false discovery rate adjustment. Missing data proportions are reported in the Supplementary Material. GCS=Glasgow Coma Scale, AIS= Abbreviated Injury Scale, ED=Emergency Department, SBP=Systolic Blood Pressure.

|  |  | **ES Status** | | |  |
| --- | --- | --- | --- | --- | --- |
| **Variable** | **Overall** N = 870^1^ | **No ES** N = 501^1^ | **LS** N = 168^1^ | **NLS** N = 201^1^ | **q**^3^ |
| **S100B [μg/L]** | 0.29 (0.18, 0.54) | 0.28 (0.17, 0.51) | 0.32 (0.20, 0.61) | 0.32 (0.20, 0.57) | **0.039** |
| Missing | 204 | 125 | 44 | 35 |  |
| **NSE [ng/mL]** | 24.37 (18.02, 34.07) | 23.18 (17.01, 33.56) | 26.37 (19.48, 39.62) | 25.09 (18.48, 33.87) | **0.017** |
| Missing | 190 | 109 | 42 | 39 |  |
| **NfL [pg/mL]** | 49.55 (24.06, 120.81) | 49.41 (22.55, 120.81) | 59.49 (36.53, 132.88) | 43.97 (24.99, 98.82) | 0.070 |
| Missing | 179 | 108 | 36 | 35 |  |
| **GFAP [pg/mL]** | 14.70 (5.60, 34.98) | 15.77 (5.79, 37.12) | 16.10 (5.97, 34.34) | 10.87 (5.05, 27.84) | 0.13 |
| Missing | 195 | 116 | 39 | 40 |  |
| **Tau [pg/mL]** | 7.51 (3.88, 15.71) | 7.24 (3.63, 15.62) | 8.44 (5.06, 16.79) | 7.23 (4.01, 14.57) | 0.2 |
| Missing | 215 | 128 | 45 | 42 |  |
| **UCH-L1 [ng/mL]** | 320.42 (161.41, 615.49) | 301.89 (139.88, 615.49) | 368.92 (201.39, 759.25) | 294.32 (187.76, 587.79) | 0.068 |
| Missing | 206 | 125 | 40 | 41 |  |

## Supplementary Table ESM2. Early Circulating Brain Injury Biomarkers According to ES Classification.

First available concentrations of S100B, NSE, GFAP, NfL, UCH-L1, and Tau following admission, stratified by extracranial surgery (ES) status: no ES, lifesaving ES (LS), and non–lifesaving ES (NLS). 1Continuous variables are reported as median (IQR). 3Group comparisons were performed using Kruskal–Wallis tests for continuous variable, with Benjamini– Hochberg false discovery rate adjustment. S100B = S100 calcium-binding protein B; NSE = Neuron specific enolase; GFAP = Glial fibrillary acidic protein; UCH-L1 = Ubiquitin C-terminal hydrolase; NfL = Neurofilament light chain.

|  |  | **ES Status** | |
| --- | --- | --- | --- |
| **Extracranial surgery type** | **Overall** N = 369^1^ | **LS** N = 168^1^ | **NLS** N = 201^1^ |
| Endovascular treatment | 10/369 (2.7%) | 10/168 (6.0%) | 0/201 (0%) |
| Extremity fracture limb (external fix) | 69/369 (19%) | 37/168 (22%) | 32/201 (16%) |
| Extremity fracture limb (internal fix) | 75/369 (20%) | 18/168 (11%) | 57/201 (28%) |
| Fasciotomy | 1/369 (0.3%) | 0/168 (0%) | 1/201 (0.5%) |
| Laparotomy (abdomen) | 23/369 (6.2%) | 21/168 (13%) | 2/201 (1.0%) |
| Maxillofacial | 38/369 (10%) | 8/168 (4.8%) | 30/201 (15%) |
| Pelvic fracture (external fix) | 11/369 (3.0%) | 10/168 (6.0%) | 1/201 (0.5%) |
| Pelvic fracture (internal fix) | 13/369 (3.5%) | 3/168 (1.8%) | 10/201 (5.0%) |
| Spinal stabilization | 46/369 (12%) | 15/168 (8.9%) | 31/201 (15%) |
| Thoracotomy | 4/369 (1.1%) | 3/168 (1.8%) | 1/201 (0.5%) |
| Wound closure/graft | 20/369 (5.4%) | 10/168 (6.0%) | 10/201 (5.0%) |
| Tracheostomy within 24h | 19 (5.1%) | 19 (11.3%) | NA |
| Other | 40/369 (11%) | 14/168 (8.3%) | 26/201 (13%) |
| **Duration ES, hours** | 2.0 (1.3, 3.2) | 2.0 (1.2, 3.3) | 2.1 (1.3, 3.2) |
| Missing | 12 | 9 | 3 |
| **Timing of ES, hours** | 21.5 (5.0, 103.8) | 6.1 (3.3, 15.3) | 67.0 (18.8, 205.8) |
| Missing | 12 | 9 | 3 |

## Supplementary Table ESM3. Type, Timing, and Duration of Extracranial Surgical Procedures.

Detailed classification of extracranial surgery (ES), including operative duration and timing from injury, stratified by lifesaving ES (LS) and non–lifesaving ES (NLS) categories. Continuous variables are presented as median (IQR); categorical variables as n/N (%).

|  |  | **ICU Discharge Status** | |
| --- | --- | --- | --- |
| **Variable** | **Overall** N = 168^1^ | **Alive** N = 147^1^ | **Dead** N = 21^1^ |
| Sex, Female | 41/168 (24.4%) | 33/147 (22.4%) | 8/21 (38.1%) |
| Age, years | 42.50 (27.00, 56.00) | 42.00 (27.00, 55.00) | 49.00 (32.00, 59.00) |
| Total ISS | 46.50 (39.50, 57.00) | 45.00 (36.00, 57.00) | 57.00 (43.00, 75.00) |
| Face (AIS≥3) | 68/168 (40.5%) | 58/147 (39.5%) | 10/21 (47.6%) |
| Extremities (AIS≥3) | 97/167 (58.1%) | 87/147 (59.2%) | 10/20 (50.0%) |
| Spine (AIS≥3) | 62/168 (36.9%) | 54/147 (36.7%) | 8/21 (38.1%) |
| Abdomen/Pelvis (AIS≥3) | 84/168 (50.0%) | 69/147 (46.9%) | 15/21 (71.4%) |
| External wounds (AIS≥3) | 16/168 (9.5%) | 11/147 (7.5%) | 5/21 (23.8%) |
| Thorax/Chest (AIS≥3) | 112/168 (66.7%) | 92/147 (62.6%) | 20/21 (95.2%) |
| GCS |  |  |  |
| 3-8 | 89/158 (56.3%) | 73/140 (52.1%) | 16/18 (88.9%) |
| 9-12 | 20/158 (12.7%) | 20/140 (14.3%) | 0/18 (0.0%) |
| 13-15 | 49/158 (31.0%) | 47/140 (33.6%) | 2/18 (11.1%) |
| Missing | 10 | 7 | 3 |
| Pupillary Reactivity |  |  |  |
| Both Reacting | 132/158 (83.5%) | 124/140 (88.6%) | 8/18 (44.4%) |
| Both unreacting | 15/158 (9.5%) | 9/140 (6.4%) | 6/18 (33.3%) |
| One Reacting | 11/158 (7.0%) | 7/140 (5.0%) | 4/18 (22.2%) |
| Missing | 10 | 7 | 3 |
| CT: Marshall scale |  |  |  |
| 1 | 18/156 (11.5%) | 17/136 (12.5%) | 1/20 (5.0%) |
| 2 | 102/156 (65.4%) | 96/136 (70.6%) | 6/20 (30.0%) |
| 3 | 15/156 (9.6%) | 6/136 (4.4%) | 9/20 (45.0%) |
| 4 | 3/156 (1.9%) | 3/136 (2.2%) | 0/20 (0.0%) |
| 5/6 | 18/156 (11.5%) | 14/136 (10.3%) | 4/20 (20.0%) |
| Missing | 12 | 11 | 1 |
| CT: Marshall scale>2 | 36/156 (23.1%) | 23/136 (16.9%) | 13/20 (65.0%) |
| ICP Monitoring (ED) | 80/168 (47.6%) | 65/147 (44.2%) | 15/21 (71.4%) |
| Hypoxemia (SpO2 < 90%) | 40/157 (25.5%) | 32/136 (23.5%) | 8/21 (38.1%) |
| Missing | 11 | 11 | 0 |
| Hypotension (SBP < 90 mmHg) | 58/158 (36.7%) | 48/137 (35.0%) | 10/21 (47.6%) |
| Missing | 10 | 10 | 0 |
| ATLS shock class |  |  |  |
| Class 1 | 97/160 (60.6%) | 89/140 (63.6%) | 8/20 (40.0%) |
| Class 2 | 15/160 (9.4%) | 14/140 (10.0%) | 1/20 (5.0%) |
| Class 3 | 7/160 (4.4%) | 6/140 (4.3%) | 1/20 (5.0%) |
| Class 4 | 41/160 (25.6%) | 31/140 (22.1%) | 10/20 (50.0%) |
| Missing | 8 | 7 | 1 |
| Base excess, mmol/L | -4.65 (-7.25, -1.45) | -3.80 (-7.00, -1.00) | -7.09 (-17.00, -5.00) |
| Missing | 60 | 49 | 11 |
| MAP At Arrival, mmHg | 86.67 (70.00, 97.50) | 87.67 (73.33, 101.33) | 72.50 (49.17, 85.50) |
| Missing | 8 | 7 | 1 |
| Blood Transfusion (ED) | 37/168 (22.0%) | 29/147 (19.7%) | 8/21 (38.1%) |
| First Glucose, mg/dL | 142.32 (119.98, 171.15) | 140.25 (117.55, 164.48) | 189.16 (147.73, 255.82) |
| Missing | 3 | 3 | 0 |
| First Haemoglobin, g/dL | 10.50 (9.20, 12.70) | 10.55 (9.29, 12.85) | 9.70 (8.90, 11.70) |
| Missing | 3 | 3 | 0 |
| First Platelets count, U/mm3 | 175.00 (126.00, 211.00) | 175.00 (130.50, 212.50) | 140.00 (97.00, 202.00) |
| Missing | 3 | 3 | 0 |
| First Fibrinogen, mg/dL | 240.00 (174.00, 340.00) | 253.00 (190.00, 354.00) | 138.00 (87.00, 189.00) |
| Missing | 27 | 24 | 3 |

## Supplementary Table ESM4. Baseline and Physiological Characteristics of lifesaving Extracranial Surgery (LS) Patients According to ICU Survival.

Injury severity, physiological parameters, imaging findings, and laboratory data among LS patients stratified by ICU survival status. 1Continuous variables are presented as median (IQR); categorical variables as n/N (%). 2Shock class was derived according to ATLS criteria; in cases of overlap, the highest severity class was assigned. ED=Emergency Department, MAP=Mean Arterial Pressure, SBP=Systolic Blood Pressure, CT=Computed Tomography, ICP = Intracranial Pressure, GCS=Glasgow Coma Scale, AIS= Abbreviated Injury Scale, Pre-Injury ASAPS=Pre-injury American Society of Anesthesiologists Physical Status, GCS=Glasgow Coma Scale, ISS=Injury Severity Score, ICU= Intensive Care Unit.

| **Parameter** | **Class I** | **Class II (Mild)** | **Class III (Moderate)** | **Class IV (Severe)** |
| --- | --- | --- | --- | --- |
| **Approximate blood loss** | <15% | 15–30% | 31–40% | >40% |
| **Heart rate** | ↔ | ↔/↑ | ↑ | ↑↑ |
| **Blood pressure** | ↔ | ↔ | ↔/↓ | ↓ |
| **Pulse pressure** | ↔ | ↓ | ↓ | ↓ |
| **Respiratory rate** | ↔ | ↔ | ↔/↑ | ↑ |
| **Urine output** | ↔ | ↔ | ↓ | ↓↓ |
| **Glasgow Coma Scale score** | ↔ | ↔ | ↓ | ↓ |
| **Base deficit^1^** | 0 to –2 mEq/L | –2 to –6 mEq/L | –6 to –10 mEq/L | –10 mEq/L or less |
| **Need for blood products** | Monitor | Possible | Yes | Massive Transfusion Protocol |

## a) ATLS classification of hypovolaemic shock 2020.

| **Parameter** | **Class I** | **Class II** | **Class III** | **Class IV** |
| --- | --- | --- | --- | --- |
| **Blood loss (% total)** | < 750 mL (<15%) | 750–1,500 mL (15–30%) | 1,500–2,000 mL (30–40%) | > 2,000 mL (>40%) |
| **Heart rate (bpm)** | Normal | > 100 | > 120 | > 140 |
| **Respiratory rate (rpm)** | Normal | 20–30 | 30–40 | > 35 |
| **Systolic blood pressure (mmHg)** | Normal | Normal | Reduced | Reduced |
| **Palpable pulse** | Radial present | Radial present | Radial not palpable | Carotid ± |
| **Neurological status** | Alert | Anxious | Confused | Lethargic |
| **Urine output (mL/h)** | Normal | 20–30 | 5–15 | Minimal |

**b) ATLS classification of hypovolaemic shock 1997.**

| **Parameter** | **Class I** | **Class II (Mild)** | **Class III (Moderate)** | **Class IV (Severe)** | **Score** |
| --- | --- | --- | --- | --- | --- |
| **Heart rate (bpm)** | ≤ 100 | 100–120 (inclusive) | 120–140 (inclusive) | > 140 | 1 |
| **Systolic blood pressure (mmHg)** | > 110 | > 110 | Any | < 110 | 1 |
| **Pulse pressure (mmHg)** | ≥ 43 | < 43 | < 43 | < 43 | 1 |
| **Respiratory rate (breaths/min)** | ≤ 20 | 20–30 (inclusive) | 30–40 (inclusive) | ≥ 35 | 1 |
| **Neurological status (GCS)** | Alert (E4, V5, M any) | Anxious (E4, V4, M any) | Confused (E4, V3, M any) | Lethargic (E ≤ 3, V < 3, M any) | 1 |
| **Base deficit (mmol/L)** | ≥ −2 | −2 to −6 | −6 to −10 | < −10 | 2 |

**c) Score system adapted to ATLS classifications fitted in the CENTER-TBI dataset.**

## Supplementary Table ESM5 (a-c). ATLS shock classification and operational criteria used for shock class derivation.

Since patients may meet criteria from multiple non–mutually exclusive classes, a score was assigned for each fulfilled criterion, and patients were classified according to the class with the highest score. In cases of equal scores, patients were assigned to the more severe class. Shock class was assigned only to patients with at least 3 of 6 available parameters.

^1^Base excess is the quantity of base (HCO3–, in mEq/L) that is above or below the normal range in the body. A negative number is called a base deficit and indicates metabolic acidosis. GCS: Glasgow coma scale. BPM: beat per minute.

|  |  | **Overall^1^** N=168 | **LS - external fixation^1^** N=47 | **Other LS procedures^1^** N=121 | **p^3^** |
| --- | --- | --- | --- | --- | --- |
| **Total ISS** |  | 47.99 (14.22) | 50.06 (13.07) | 47.19 (14.62) | 0.241 |
| **Face (AIS≥3)** |  | 68 (40.5) | 17 (36.2) | 51 (42.1) | 0.594 |
| **Spine^2^ (AIS≥3)** |  | 62 (36.9) | 9 (19.1) | 53 (43.8) | 0.005 |
| **Abdomen/Pelvis (AIS≥3)** |  | 84 (50.0) | 29 (61.7) | 55 (45.5) | 0.086 |
| **Extremities (AIS≥3)** |  | 16 (9.5) | 6 (12.8) | 10 (8.3) | 0.549 |
| **Thorax/Chest** |  | 112 (66.7) | 28 (59.6) | 84 (69.4) | 0.302 |
| **Hypotension (SBP < 90 mmHg)** |  | 58 (36.7) | 14 (31.1) | 44 (38.9) | 0.460 |
| **ATLS shock class^4^ (%)** | Class 1 | 97 (60.6) | 28 (60.9) | 69 (60.5) | 0.328 |
|  | Class 2 | 15 (9.4) | 7 (15.2) | 8 (7.0) |  |
|  | Class 3 | 7 (4.4) | 1 (2.2) | 6 (5.3) |  |
|  | Class 4 | 41 (25.6) | 10 (21.7) | 31 (27.2) |  |
| **MAP At Arrival, mmHg** |  | 85.28 (20.88) | 86.43 (21.69) | 84.82 (20.64) | 0.663 |
| **Base excess, mmol/L** |  | -5.06 (5.77) | -4.62 (6.01) | -5.21 (5.72) | 0.640 |
| **First Haemoglobin, g/dL** |  | 10.88 (2.23) | 10.63 (2.24) | 10.97 (2.23) | 0.393 |
| **First Fibrinogen, mg/dL** |  | 208.35 (101.90) | 191.68 (88.92) | 214.49 (106.03) | 0.240 |
| **Coagulopathy Correction (ED)** |  | 80 (48.2) | 29 (61.7) | 51 (42.9) | 0.044 |
| **Lactate (mmol/L)** |  | 4.75 (9.48) | 3.27 (3.14) | 5.27 (10.84) | 0.348 |
| **RBC Transfusion** |  | 60 (75.0) | 22 (75.9) | 38 (74.5) | 1.000 |
| **Vasopressors** |  | 0.20 (0.40) | 0.26 (0.44) | 0.18 (0.39) | 0.290 |
| **6-mo mortality GOSE=1** |  | 26 (15.5) | 7 (14.9) | 19 (15.7) | 1.000 |
| **6-mo GOSE <=4** |  | 87 (51.8) | 28 (59.6) | 59 (48.8) | 0.277 |

## Supplementary Table ESM6. Baseline and physiological characteristics of LS external fixation versus other LS procedures.

^1^Continuous variables are presented as median (IQR); categorical variables as n/N (%). ^2^Includes cervical spine, thoracic spine, lumbar spine. ^3^Group comparisons were performed using Kruskal–Wallis tests for continuous variables and χ² tests for categorical variables, with Benjamini– Hochberg false discovery rate adjustment. Missing data proportions are reported in the Supplementary Material. GCS=Glasgow Coma Scale, AIS= Abbreviated Injury Scale, ED=Emergency Department, SBP=Systolic Blood Pressure. ^4^Shock class was derived according to ATLS criteria; in cases of overlap, the highest severity class was assigned.

## Supplementary Figure ESM1: Flowchart.

Group cohort selection: ES = extracranial surgery, LS = ES lifesaving (*patients underwent tracheostomy within 24h were included in this group), NLS = ES non-lifesaving.

## Supplementary Figure ESM2: Upset plots.

## Upset plots of severe injuries (AIS ≥3) by body region and surgery group. Left bars: total patients per region. Vertical bars: patients with injuries in the combinations shown by connected dots. Top: ES vs. No-ES. Bottom: LS vs. NLS. ES = extracranial surgery, No-ES = no extracranial surgery, LS = ES lifesaving, NLS = ES non-lifesaving.

## Supplementary Figure ESM3. Subgroup Analysis of life-saving Extracranial Surgery (LS) Patients Stratified by ICU Survival.

## Boxplots showing the distribution of clinical and biomarker parameters in LS patients according to ICU survival status (alive vs dead). Survivors are represented by green boxes, whereas non-survivors are shown in red. Values are presented as boxplots with the median indicated. DBP = diastolic blood pressure; HR = heart rate; SBP = systolic blood pressure; S100B = S100 calcium-binding protein B; NSE = neuron-specific enolase; GFAP = glial fibrillary acidic protein; UCH-L1 = ubiquitin C-terminal hydrolase-L1; NfL = neurofilament light chain. ICU = Intensive Care Unit.
